# Effects of surgery versus radiotherapy in patients with localized prostate cancer in terms of urinary, bowel, and sexual domains

**DOI:** 10.1002/cam4.6395

**Published:** 2023-07-30

**Authors:** Chao Yu, Jie Yao, Yujing He, Jianing Huang, Meiling Chen, Mingxia Qian, Dandi Lou, Zhizhen Zhou, Feng Chen

**Affiliations:** ^1^ Department of Urology, Ningbo Medical Center LiHuiLi Hospital Ningbo University Ningbo China; ^2^ School of Public Health Zhejiang Chinese Medical University Hangzhou China; ^3^ The Second Clinical Medical College Zhejiang Chinese Medical University Hangzhou China; ^4^ School of Public Health Shanghai Jiao Tong University School of Medicine Shanghai China; ^5^ The First Clinical Medical College Zhejiang Chinese Medical University Hangzhou China; ^6^ Urology Department Ningbo Yinzhou No. 2 Hospital Ningbo China

**Keywords:** health‐related quality of life, prostate cancer, radiotherapy, surgery

## Abstract

**Background:**

The health‐related quality of life (HRQoL) of patients with localized prostate cancer (LPCa) after treatment mainly surgery and radiotherapy (RT) has received increasing attention. The aim of this study is to compare the HRQoL of LPCa after surgery and RT.

**Methods:**

Web of Science, Embase, PubMed and Cochrane databases were searched after January 2000 to observe the HRQoL scores after surgery and RT at different treatment time points.

**Results:**

A total of 28 studies were included in this study, and the results showed that LPCa received surgery had better bowel scores than RT at ≤3 (weighted mean differences [WMD] = 4.18; *p* = 0.03), 3–6 (WMD = 4.16; *p* < 0.001), 6–12 (WMD = 2.99; *p* = 0.004), 24–60 (WMD = 1.87; *p* = 0.06), and ≥60 (WMD = 4.54; *p* = 0.02) months. However, LPCa received RT had higher urinary scores at ≤3 (WMD = −7.39; *p* = 0.02), 3–6 (WMD = −6.03; *p* = 0.02), 6–12 (WMD = −4.90; *p* < 0.001), 24–60 (WMD = −3.96; *p* < 0.001), ≥60 (WMD = −2.95; *p* < 0.001) months and had better sexual scores at ≤3 (WMD = −13.58; *p* = 0.09), 3–6 (WMD = −12.32; *p* = 0.06), 6–12 (WMD = −12.03; *p* = 0.002), 24–60 (WMD = −11.29; *p* < 0.001), and ≥60 (WMD = −3.10; *p* = 0.46) months than surgery. The scores difference between surgery and RT decreased over time.

**Conclusion:**

Overall, for LPCa, surgery was associated with better HRQoL in the bowel domain, whereas RT was associated with better HRQoL in the urinary and sexual domains, with the difference between surgery and RT narrowing over time.

## 1 INTRODUCTION

Prostate cancer is the second most common cancer in men worldwide and the fifth leading cause of cancer death in men,^1^ with localized prostate cancer (LPCa) accounting for the majority of all stages of prostate cancer.^2^ At present, the main treatments for LPCa are surgery, radiotherapy (RT) and active surveillance. Studies^3^, ^4^ have shown that there was no significant difference in long‐term survival (cancer‐specific mortality and overall mortality) between the two groups, and that the treatment outcomes were good for both. However, the health‐related quality of life (HRQoL) of post‐treatment patients was quite poor, especially in the urinary, bowel, and sexual function.^5^, ^6^, ^7^ Therefore, optimizing the HRQoL was one of the important research directions in the treatment of LPCa. The most important aspects of HRQoL are urinary, bowel, and sexual domains. Some studies have also debated the impact of surgery and RT on the HRQoL in LPCa.^8^, ^9^, ^10^, ^11^ With the progress of medical technology, surgical approaches and RT patterns of LPCa have also changed.^12^, ^13^, ^14^, ^15^, ^16^, ^17^, ^18^, ^19^ For example, RT modes represented by intensity‐modulated radiation therapy and surgical approaches represented by robotic radical prostatectomy have achieved more precise treatment and better HRQoL than previous medical techniques. Therefore, this meta‐analysis brings together studies published since January 2000 to assess the differences in LPCa between surgery and RT in terms of urinary, bowel, and sexual aspects.

## 2 MATERIALS AND METHODS

### 2.1 Search strategy

This meta‐analysis searched Web of Science, PubMed, Cochrane and Embase databases from January 2000 to January 2023 for English‐language studies reporting HRQoL differences between surgery and RT for LPCa. The search was dominated by the following search terms (“EBRT” OR “External beam radiation therapy” OR “brachytherapy” OR “BT” OR “Radiotherapy”) AND (“Locally advanced prostate cancer” OR “LAPC” OR “Prostatic Neoplasms”) AND (“Surgery” OR “operations” OR “Prostatectomy”). Detailed search strategies are provided in Table S1. In addition, the references of the included articles were further searched. The screening of the literature studies was completed independently by two researchers. This meta‐analysis has been registered with PROSPERO under registration number CRD42023405334.

### 2.2 Study selection

The inclusion criteria for this meta‐analysis were as follows: (1) randomized controlled trials (RCTs), cohort studies or case–control studies published after January 2000, including retrospective studies; (2) men reported in the study were diagnosed with LPCa; (3) the experimental group was treated with surgery, and the control group with RT, HRQoL was reported but not limited to urinary, bowel, or sexual aspects; (4) there were uniform instruments to assess HRQoL between the two groups; (5) mean and standard deviation of HRQoL outcome scores data were presented or computable.

The exclusion criteria involved are as follows: (1) The time at which HRQoL assessments were provided was unclear and (2) data could not be calculated. When the same clinical trial was updated and published, the most recently published study or the study reporting more comprehensive data was selected.

### 2.3 Data extraction

Two researchers extracted data independently, and in case of inconsistent results, a third researcher verified the data to ensure the accuracy. The following information was extracted from the study: author, year, country, follow‐up time, number of patients undergoing radical prostatectomy (RP) and RT, HRQoL assessment scale, HRQoL outcomes. In terms of functional outcomes, there were three major components: urinary, bowel, and sexual. The urinary related outcomes included urinary summary, urinary function, urinary bother, urinary incontinence, and urinary irritative/obstructive; the bowel related outcomes included bowel, bowel function, and bowel bother; the sexual related outcomes included sexual, sexual function, and sexual bother.

The common instruments for the assessment of urinary, bowel, and sexual function are the scaled University of California Los Angeles Prostate Cancer Index (UCLA PCI) and the Expanded Prostate Cancer Index Composite (EPIC), with total scores ranging from 0 to 100, with higher scores indicating better overall function of the system. The study nodes of HRQoL reported were divided into ≤3 months, 3–6 months, 6–12 months, 24–60 months, and ≥60 months.

### 2.4 Statistical analysis

We used Review Manager (RevMan) 5.3 software for statistical analysis. Heterogeneity between articles was measured by *p*‐value of chi‐squared test and *I* ^2^. The null hypothesis was that there was no heterogeneity between studies, and the null hypothesis was rejected when *p* < 0.1 and considered that there was heterogeneity between studies. Heterogeneity was considered low when *I* ^2^ was below 25%, moderate between 25% and 50%, high between 50% and 75%, and extremely high when *I* ^2^ was above 75%. Random‐effects models were performed for all statistical analyses to ensure the reliability of this study. Weighted mean differences (WMD) and 95% confidence intervals (CI) were used to assess continuous variables. When WMD <0, the HRQoL of the LPCa receiving RT was superior to that of those receiving surgery. Conversely, when WMD >0, it means that LPCa patients who underwent surgery had a better HRQoL than those underwent RT. The difference was statistically significant when the *p*‐value was <0.05. Some studies chose sample median, first and third quartile instead of sample mean and standard deviation. For these studies, when included in the meta analysis, the estimation method of Luo and Wan^20^, ^21^, ^22^, ^23^ was usually applied to transform the data back to the sample mean and standard deviation.

Table S2 shows the quality assessment of the included studies. After assessment of the studies by the Cochrane Collaboration's tool and Newcastle–Ottawa Quality Assessment Scale's, we found that all 27 observational studies scored 6 or higher, and the only RCT was of high quality. The overall quality met the requirements of this meta‐analysis.

## 3 RESULTS

### 3.1 Literature search

**FIGURE 1:**
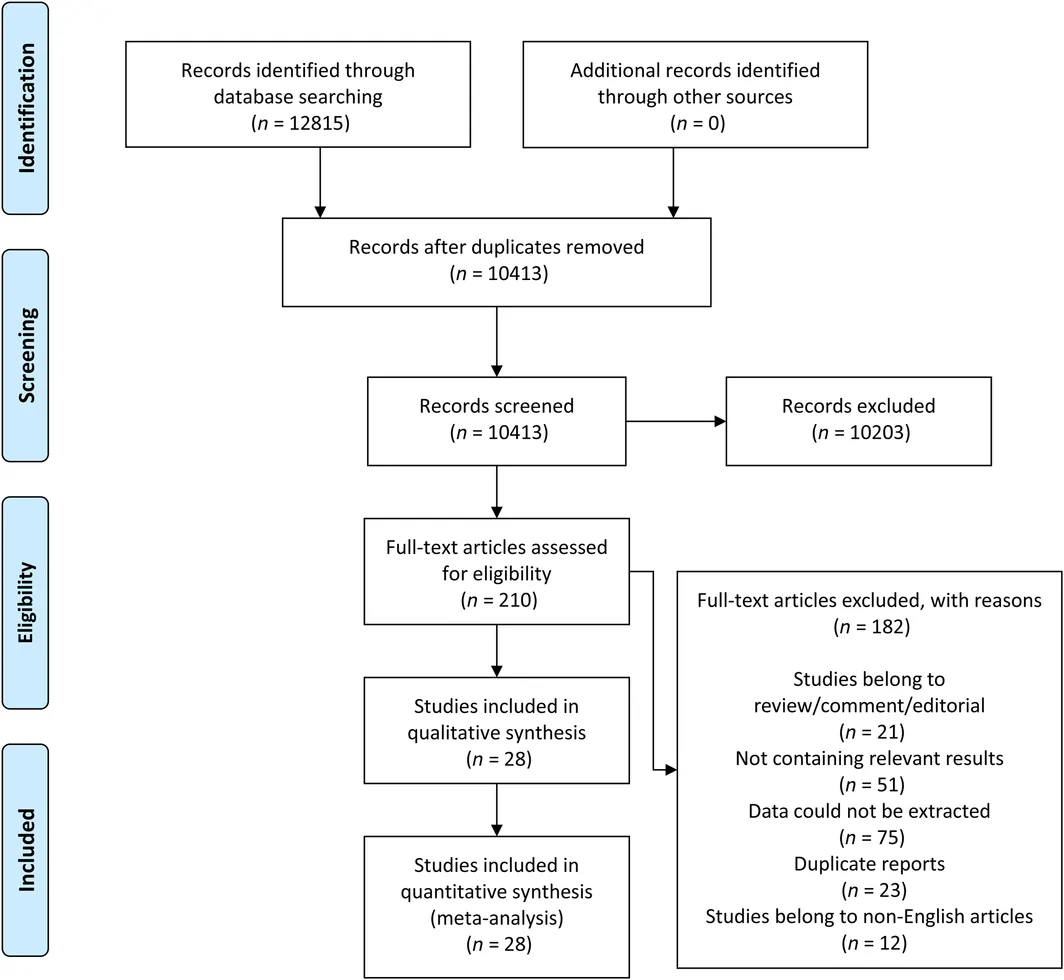
A schematic flowchart for the selection of articles included in this meta‐analysis.

Figure 1 shows the literature search and screening process for this meta‐analysis. A preliminary search of four databases, namely, Web of Science, PubMed, Cochrane, and Embase, identified 12,815 studies. After excluding 2402 duplicate articles and 10,203 articles with non‐compliant titles and abstracts, 210 articles remained. Among the remaining 210 studies, 28 studies^8^, ^9^, ^10^, ^11^, ^24^, ^25^, ^26^, ^27^, ^28^, ^29^, ^30^, ^31^, ^32^, ^33^, ^34^, ^35^, ^36^, ^37^, ^38^, ^39^, ^40^, ^41^, ^42^, ^43^, ^44^, ^45^, ^46^, ^47^ ultimately met the inclusion criteria.

### 3.2 Characteristics of included studies

A total of 28 studies (published between January 2000 and January 2023) were included in this meta‐analysis, of which 10 were from North America, six were from Europe, nine were from Asia, and the remaining three were from Australia. Except for one study^9^ which was an RCT, the remaining 27 studies were observational studies. All studies were scored from 0 to 100 using either UCLA PCI or EPIC to measure urinary, bowel, and sexual domains. Additional details are provided in Table 1. Table S3 documents the data sources of each study and the methods and doses of RT.

**TABLE 1:** Characteristics of included studies in the meta‐analysis of the difference of HRQoL between surgery and radiotherapy for LPCa.

Author, year	Country	Follow‐up time (month)	Patient numbers		Assessment of HRQoL outcomes	HRQoL domain
			Radical prostatectomy	Radiotherapy		
Downs TM, 2003	USA	24	327	BT: 92	UCLA PCI	Urinary function; Urinary bother; Bowel function; Bowel bother; Sexual function; Sexual bother
Namiki S, 2004	Japan	12	186	EBRT: 78	UCLA PCI	Urinary function; Urinary bother; Bowel function; Bowel bother; Sexual function; Sexual bother
Namiki S, 2006	Japan	12	67	BT: 70	UCLA PCI	Urinary function; Urinary bother; Bowel function; Bowel bother; Sexual function; Sexual bother
Namiki S, 2010	Japan	24	166	EBRT: 118	UCLA PCI	Urinary function; Urinary bother; Bowel function; Bowel bother; Sexual function; Sexual bother
Miller DC, 2005	USA	72	665	EBRT: 147 BT: 84	EPIC‐26	Urinary irritative; Urinary incontinence; Bowel; Sexual
Symon Z, 2006	USA	12	24	EBRT: 26	EPIC	Urinary irritative; Urinary incontinence; Bowel; Sexual
Korfage IJ, 2005	Netherlands	52	127	EBRT: 187	UCLA PCI	Urinary function; Urinary function; Bowel function; Bowel bother
Jayadevappa R, 2006	USA	12	69	EBRT: 46	UCLA PCI	Urinary function; Urinary bother; Bowel function; Bowel bother; Sexual function; Sexual bother
Hashine K, 2008	Japan	12	122	BT: 82	UCLA PCI	Urinary function; Urinary bother; Bowel function; Bowel bother; Sexual function; Sexual bother
Hashine K, 2009	Japan	12	96	BT: 88	EPIC	Urinary function; Urinary incontinence; Urinary irritative/obstructive; Urinary bother; Bowel function; Bowel bother; Sexual function; Sexual bother
Ferrer M, 2008	Spain	24	134	EBRT: 205 BT: 275	EPIC	Urinary; Bowel; Sexual
Krahn MD, 2009	Canada	12	68	66	UCLA PCI	Urinary function; Urinary bother; Bowel function; Bowel bother; Sexual function; Sexual bother
Takizawa I, 2009	Japan	36	86	EBRT: 76	UCLA PCI	Urinary function; Urinary bother; Bowel function; Bowel bother; Sexual function; Sexual bother
Smith DP, 2009	Australia	36	970	EBRT: 123 BT: 47	UCLA PCI	Urinary function; Urinary bother; Bowel function; Bowel bother; Sexual function; Sexual bother
Egger SJ, 2018	Australia	120	221	BT: 32	EPIC‐26	Urinary incontinence; Urinary bother; Sexual; Sexual bother; Bowel bother
Rice K, 2010	USA	12	370	EBRT: 154 BT: 28	EPIC	Urinary function; Urinary bother; Bowel function; Bowel bother; Sexual function; Sexual bother
Dragićević S, 2010	Serbia	12	96	BT: 88	EPIC	Urinary function; Urinary incontinence; Urinary irritative/obstructive; Urinary bother; Bowel function; Bowel bother; Sexual function; Sexual bother
Crook JM, 2011	Canada	60	66	BT: 102	EPIC	Urinary; Bowel; Sexual
van Tol‐Geerdink JJ, 2013	Netherlands	12	143	EBRT: 36 BT: 25	EPIC	Urinary; Urinary function; Urinary bother; Urinary incontinence; Bowel; Bowel function; Bowel bother; Sexual; Sexual function; Sexual bother
Shinohara N, 2013	Japan	60	48	EBRT: 23	UCLA PCI	Urinary function; Urinary bother; Bowel function; Bowel bother; Sexual function; Sexual bother
Donovan JL, 2016	UK	72	553	EBRT: 545	EPIC	Urinary; Urinary incontinence; Urinary bother; Urinary irritative/obstructive; Bowel; Bowel function; Bowel bother; Sexual; Sexual function; Sexual bother
Chang P, 2017	USA	24	522	EBRT: 239 BT: 260	EPIC‐26	Urinary incontinence; Urinary irritative/obstructive
Sciarra A, 2018	Italy	12	99	EBRT: 102	UCLA PCI	Urinary; Bowel; Sexual
Hoffman KE, 2020	USA	60	675	EBRT: 261 BT: 87	EPIC‐26	Urinary incontinence; Urinary irritative; Bowel function; Sexual function
Ng CF, 2020	China	72	30	EBRT: 63	EPIC	Urinary; Urinary function; Urinary bother; Urinary incontinence; Urinary irritative/obstructive; Bowel; Bowel function; Bowel bother; Sexual; Sexual function; Sexual bother
Wang F, 2022	China	24	312	BT: 245	EPIC	Urinary function; Urinary bother; Urinary incontinence; Urinary irritative/obstructive; Bowel function; Bowel bother; Sexual function; Sexual bother
Tiruye T, 2022	Australia	12	2810	EBRT: 1011 BT: 343	EPIC‐26	Urinary incontinence; Urinary obstructive; Bowel function; Sexual function
Taylor KL, 2012	USA	90	201	110	EPIC	Urinary function; Bowel bother; Sexual function

Abbreviations: BT, brachytherapy; EBRT, external beam radiotherapy; EPIC, Expanded Prostate Cancer Index Composite; HRQoL, health‐related quality of life; LPCa, localized prostate cancer; RCT, randomized controlled trial; UK, United Kingdom; USA, United States of America; UCLA PCI, the University of California, Los Angeles Prostate Cancer Index.

### 3.3 Urinary HRQoL

Table 2 showed the difference in urinary scores, urinary function scores, and urinary bother scores between LPCa patients undergoing surgery and RT. In terms of urinary scores, the results were reported for different time periods were as follows: ≤3 (WMD = −7.39, 95% CI = [−13.82, −0.97], *p* = 0.02), 3–6 (WMD = −6.03, 95% CI = [−10.93, −1.12], *p* = 0.02), 6–12 (WMD = −4.90, 95% CI = [−7.06, −2.74], *p* < 0.001), 24–60 (WMD = −3.96, 95% CI = [−5.87, −2.06], *p* < 0.001), and ≥60 (WMD = −2.95, 95% CI = [−4.18, −1.73], *p* < 0.001) months after the corresponding treatment, the results all showed that the urinary scores of LPCa who underwent surgery were lower than those who received RT. In terms of urinary function scores, the following results were reported for different time periods: ≤3 (WMD = −20.40, 95% CI = [−26.08, −14.73], *p* < 0.001), 3–6 (WMD = −14.27, 95% CI = [−17.93, −10.61], *p* < 0.001;), 6–12 (WMD = −11.00, 95% CI = [−13.46, −8.54], *p* < 0.001), 24–60 (WMD = −8.70, 95% CI = [−12.18, −5.22], *p* < 0.001), and ≥60 (WMD = −15.04, 95% CI = [−19.61, −10.48], *p* < 0.001) months after corresponding treatment. The results also showed that the urinary function scores of LPCa patients who received surgery were lower than those who underwent RT. Notably, LPCa patients who underwent RT showed a roughly decreasing trend over time in terms of the advantages of voiding scored and voiding function scores compared with surgery. However, there was no statistically significant difference in urinary bother scores between LPCa patients who received surgery or RT at each period (≤3, 3–6, 6–12, 24–60, ≥60 months).

**TABLE 2:** Analysis of the difference of urinary between surgery and radiotherapy for LPCa.

Report time (month)	No. of studies	WMD	95% CI	*p*	Heterogeneity (*I* ^2^) (%)
Urinary scores					
≤3	2	−7.39	−13.82, −0.97	0.02	94
3–6	3	−6.03	−10.93, −1.12	0.02	96
6–12	4	−4.90	−7.06, −2.74	<0.001	84
24–60	2	−3.96	−5.87, −2.06	<0.001	48
≥60	10	−2.95	−4.18, −1.73	<0.001	0
Urinary function scores					
≤3	10	−20.40	−26.08, −14.73	<0.001	93
3–6	11	−14.27	−17.93, −10.61	<0.001	89
6–12	14	−11.00	−13.46, −8.54	<0.001	79
24–60	6	−8.70	−12.18, −5.22	<0.001	85
≥60	3	−15.04	−19.61, −10.48	<0.001	0
Urinary bother scores					
≤3	10	−0.57	−5.28, 4.14	0.81	90
3–6	12	1.00	−1.71, 3.71	0.47	79
6–12	15	−0.67	−2.52, 1.18	0.48	68
24–60	7	−0.12	−1.89, 1.66	0.9	38
≥60	4	−0.63	−2.09, 0.83	0.4	0

Abbreviations: CI, confidence interval; LPCa, localized prostate cancer; WMD, weighted mean difference.

Table 3 showed the differences in urinary incontinence scores and urinary irritability or obstructivity scores between LPCa patients who underwent surgery or RT. LPCa who received RT had better urinary incontinence scores than those who underwent surgery at ≤3 (WMD = −24.87, 95% CI = [−27.09, −22.65], *p* < 0.001), 3–6 (WMD = −16.79, 95% CI = [−19.82, −13.77], *p* < 0.001), 6–12 (WMD = −13.63, 95% CI = [−15.38, −11.87], *p* < 0.001), 24–60 (WMD = −8.82, 95% CI = [−12.74, −4.90], *p* < 0.001), and ≥60 (WMD = −10.17, 95% CI = [−14.50, −5.84], *p* < 0.001) months, respectively. However, during ≤3 (WMD = 6.81, 95% CI = [3.78, 9.84], *p* < 0.001), 3–6 (WMD = 6.41, 95% CI = [3.89, 8.93], *p* < 0.001), 6–12 (WMD = 4.37, 95% CI = [2.34, 6.40], *p* < 0.001), 24–60 (WMD = 2.86, 95% CI = [0.23, 5.48], *p* = 0.03), and ≥60 (WMD = 4.34, 95% CI = [1.20, 7.48], *p* = 0.007) months, respectively, those who reported receiving RT had lower urinary irritability or obstructivity scores than in those undergoing surgery. It was worth mentioning that the difference between LPCa patients who underwent surgery and those who received RT also generally decreased in these two scores.

**TABLE 3:** Analysis of the difference of urinary between surgery and radiotherapy for LPCa.

Report time (month)	No. of studies	WMD	95% CI	*p*	Heterogeneity (*I* ^2^) (%)
Urinary incontinence scores					
≤3	3	−24.87	−27.09, −22.65	<0.001	0
3–6	5	−16.79	−19.82, −13.77	<0.001	73
6–12	8	−13.63	−15.38, −11.87	<0.001	42
24–60	4	−8.82	−12.74, −4.90	<0.001	92
≥60	5	−10.17	−14.50, −5.84	<0.001	84
Urinary irritative or obstructive scores					
≤3	3	6.81	3.78, 9.84	<0.001	68
3–6	5	6.41	3.89, 8.93	<0.001	87
6–12	8	4.37	2.34, 6.40	<0.001	87
24–60	4	2.86	0.23, 5.48	0.03	92
≥60	4	4.34	1.20, 7.48	0.007	88

Abbreviations: CI, confidence interval; LPCa, localized prostate cancer; WMD, weighted mean difference.

### 3.4 Bowel HRQoL

Table 4 showed the differences in scores between surgery and RT in terms of bowel. The results showed that the bowel scores of LPCa patients who received surgery were higher than those who underwent RT at ≤3 (WMD = 4.18, 95% CI = [0.45, 7.92], *p* = 0.03), 3–6 (WMD = 4.16, 95% CI = [2.34, 5.98], *p* < 0.001), 6–12 (WMD = 2.99, 95% CI = [0.95, 5.03], *p* = 0.004), 24–60 (WMD = 1.87, 95% CI = [−0.09, 3.84], *p* = 0.06), and ≥60 (WMD = 4.54, 95% CI = [0.60, 8.47], *p* = 0.02) months, and the differences were generally progressively reduced. The results all showed that LPCa patients who received surgery had higher bowel function scores than those who received RT at ≤3 (WMD = 2.73, 95% CI = [0.66, 4.79], *p* = 0.01), 3–6 (WMD = 4.21, 95% CI = [2.65, 5.78], *p* < 0.001), 6–12 (WMD = 2.61, 95% CI = [1.30, 3.91], *p* < 0.001), 24–60 (WMD = 2.17, 95% CI = [0.52, 3.83], *p* = 0.01), and ≥60 (WMD = 2.56, 95% CI = [0.91, 4.22], *p* = 0.002) months. In addition, the periods at ≤3 (WMD = 4.48, 95% CI = [2.32, 6.64], *p* < 0.001), 3–6 (WMD = 4.26, 95% CI = [2.14, 6.38], *p* < 0.001), 6–12 (WMD = 3.60, 95% CI = [1.72, 5.48], *p* < 0.001), 24–60 (WMD = 5.57, 95% CI = [2.50, 8.64], *p* < 0.001), and ≥60 (WMD = 2.43, 95% CI = [0.98, 3.87], *p* = 0.001) months showed that those who underwent surgery had higher bowel bother scores than those who underwent RT.

**TABLE 4:** Analysis of the difference of bowel between surgery and radiotherapy for LPCa.

Report time (month)	No. of studies	WMD	95% CI	*p*	Heterogeneity (*I* ^2^) (%)
Bowel scores					
≤3	2	4.18	0.45, 7.92	0.03	96
3–6	3	4.16	2.34, 5.98	<0.001	79
6–12	5	2.99	0.95, 5.03	0.004	87
24–60	2	1.87	−0.09, 3.84	0.06	77
≥60	4	4.54	0.60, 8.47	0.02	88
Bowel function scores					
≤3	10	2.73	0.66, 4.79	0.01	73
3–6	13	4.21	2.65, 5.78	<0.001	81
6–12	17	2.61	1.30, 3.91	<0.001	80
24–60	7	2.17	0.52, 3.83	0.01	82
≥60	4	2.56	0.91, 4.22	0.002	61
Bowel bother scores					
≤3	10	4.48	2.32, 6.64	<0.001	76
3–6	12	4.26	2.14, 6.38	<0.001	86
6–12	15	3.60	1.72, 5.48	<0.001	84
24–60	7	5.57	2.50, 8.64	<0.001	86
≥60	5	2.43	0.98, 3.87	0.001	0

Abbreviations: CI, confidence interval; LPCa, localized prostate cancer; WMD, weighted mean difference.

### 3.5 Sexual HRQoL

Table 5 showed the difference in sexual differences between surgery and RT. The period at ≤3 (WMD = −13.58, 95% CI = [−29.19, 2.03], *p* = 0.09), 3–6 (WMD = −12.32, 95% CI = [−25.37, 0.73], *p* = 0.06), 6–12 (WMD = −12.03, 95% CI = [−19.66, −4.40], *p* = 0.002), 24–60 (WMD = −11.29, 95% CI = [−16.85, −5.74], *p* < 0.001), and ≥60 (WMD = −3.10, 95% CI = [−11.29, 5.09], *p* = 0.46) months. It approximately showed that LPCa patients who underwent RT had higher sexual scores than those who underwent surgery, with statistically significant differences at 6–12, and 24–60 months. At approximately all time periods, the sexual function scores for RT in LPCa patients were higher than those of surgery at ≤3 (WMD = −14.64, 95% CI = [−19.92, −9.37], *p* < 0.001), 3–6 (WMD = −15.21, 95% CI = [−20.85, −9.58], *p* < 0.001), 6–12 (WMD = −11.53, 95% CI = [−15.20, −7.87], *p* < 0.001), 24–60 (WMD = −2.39, 95% CI = [−6.00, 1.22], *p* = 0.19), and ≥60 (WMD = −2.79, 95% CI = [−12.04, 6.46], *p* = 0.55) months, with statistically significant differences at ≤3, 3–6, 6–12 months. In terms of sexual bother scores, ≤3 (WMD = −16.18, 95% CI = [−20.50, −11.85], *p* < 0.001), 3–6 (WMD = −16.48, 95% CI = [−21.50, −11.46], *p* < 0.001), 6–12 (WMD = −15.34, 95% CI = [−18.73, −11.94], *p* < 0.001), 24–60 (WMD = −9.56, 95% CI = [−14.03, −5.09], *p* < 0.001), and ≥60 (WMD = −8.04, 95% CI = [−12.13, −3.94], *p* < 0.001) months all showed that the sexual bother scores of the LPCa patients who received RT were higher than those who received surgery, and the difference was statistically significant. Over time, the difference between the advantages of RT and surgery in terms of sexual summary, sexual function, and sexual bother scores of LPCa patients gradually narrowed.

**TABLE 5:** Analysis of the difference of sexual between surgery and radiotherapy for LPCa.

Report time (month)	No. of studies	WMD	95% CI	*p*	Heterogeneity (*I* ^2^) (%)
Sexual scores					
≤3	2	−13.58	−29.19, 2.03	0.09	98
3–6	3	−12.32	−25.37, 0.73	0.06	98
6–12	5	−12.03	−19.66, −4.40	0.002	95
24–60	2	−11.29	−16.85, −5.74	<0.001	77
≥60	5	−3.10	−11.29, 5.09	0.46	87
Sexual function scores					
≤3	10	−14.64	−19.92, −9.37	<0.001	94
3–6	12	−15.21	−20.85, −9.58	<0.001	95
6–12	16	−11.53	−15.20, −7.87	<0.001	89
24–60	7	−2.39	−6.00, 1.22	0.19	78
≥60	5	−2.79	−12.04, 6.46	0.55	85
Sexual bother scores					
≤3	10	−16.18	−20.50, −11.85	<0.001	70
3–6	11	−16.48	−21.50, −11.46	<0.001	83
6–12	14	−15.34	−18.73, −11.94	<0.001	66
24–60	6	−9.56	−14.03, −5.09	<0.001	67
≥60	4	−8.04	−12.13, −3.94	<0.001	0

Abbreviations: CI, confidence interval; LPCa, localized prostate cancer; WMD, weighted mean difference.

## 4 DISCUSSION

The clinical studies included in this meta‐analysis assessed HRQoL in LPCa patients treated with RT or surgery using either the UCLA‐PCI or the EPIC scale. The EPIC scale was an updated version of the UCLA‐PCI scale. The EPIC includes more questions about urinary and bowel function, such as urinary and bowel irritation. In contrast, UCLA‐PCI does not include rectal bleeding and anaphylaxis, and the main component of urinary domain was urinary incontinence.^48^ The scale had internal consistency and reliability in the scores for both function and intrusion. The results of this study showed that surgery achieved better results in terms of urinary irritative or obstructive subscores, which might be a factor to consider the obstructive symptoms before the LPCa treatment. This could be explained by RT‐induced atrophy of the prostatic glandular epithelium, interstitial fibrosis, and vascular changes (endomysial hyperplasia with luminal narrowing), resulting in delayed epithelialization with constant exposure of the submucosa to urine, persistent irritation of nerve endings, stone formation, infection, granulation tissue, and accumulation of fibrin, all of which may block the lumen.^49^, ^50^

The total urinary score, urinary function score, and urinary incontinence score of surgery were poor, and the related mechanisms were discussed as follows. Above all, it is well known that the urethral sphincter complex controls urination and consists of a smooth internal sphincter for passive continence and a skeletal external sphincter for active continence.^51^ Prostatectomy often impairs the structure of the bladder neck, reduction in the internal sphincter can result in urinary incontinence.^52^, ^53^, ^54^ Furthermore, the neurovascular bundles and related nerves (pudendal and sympathetic nerves) innervate the membranous urethra, and the surgery was often accompanied by lymph node dissection, which means that prostatectomy had a high probability of damaging related nerves and affecting the mechanism of urinary control.^55^, ^56^, ^57^, ^58^ Considering the distribution of the autonomic nerves around the posterior proximal urethra and the bladder neck, surgical damage to the nerves that control detrusor contraction will lead to reduced bladder contraction and urinary dysfunction.^59^, ^60^, ^61^, ^62^ In addition, the male urethral support structure composed of the anterior and posterior support structures and the pelvic floor support structure played an all‐round role in suspension support and stabilization of the urethral sphincter complex.^63^, ^64^, ^65^, ^66^, ^67^, ^68^ The anatomical stability of urethral sphincter contributes to urinary control,^69^, ^70^, ^71^, ^72^, ^73^ for example, direct injury to the pubopudendal muscle and puboprostatic ligament could occur after extensive dissection of the apex of the prostate; urethral vesical anastomosis sutures in the lateral tissue may also damage the puboperineal muscle and eventually cause incontinence. On the side, postoperative overactivity of the detrusor and shortening of urethral length postoperatively could also contribute to incontinence,^74^, ^75^ which occurs when the relative stiffness of the reconstructed urethra is insufficient and the biomechanical response is impaired.^76^ However, RT caused much less damage to the surrounding muscles and nerves.^77^, ^78^

As for sexual function, RP had been shown to cause erectile dysfunction by damaging the pelvic plexus, which provides autonomic innervation to the corpus cavernosum.^57^, ^79^ In addition, the surgical procedure is usually accompanied by extensive dissection of lymph nodes, including the internal iliac region and the common iliac region. The removal of the internal iliac lymph nodes may cause damage to the inferior gastric plexus, which carries sympathetic and cholinergic nerves, resulting in erectile dysfunction, ejaculation and bladder neck dysfunction.^80^, ^81^ This was consistent with the results of the present study showing worse sexual domains after surgery. Nevertheless, the damage of RT to sexual domain was relatively small.^82^

In the bowel field, our study showed that surgery was superior to RT, which could be explained by the proximity of the prostate to the rectum, whereas the toxic effect of RT on the intestine could lead to fibrosis, destruction of the anal sphincter nerve plexus and rectal inhibitory reflex, mainly manifested as diarrhea and fecal incontinence.^83^, ^84^ Other studies had shown that RT could reduce anal resting pressure and rectal distensibility and often cause rectal mucosal telangiectasia. Objective changes might be associated with fecal incontinence, frequent bowel movements, urgency and rectal bleeding.^85^ However, surgical resection of the whole tumor has little effect on bowel function.

In a word, HRQoL in the urinary, sexual and bowel domains in LPCa patients differ significantly between surgery and RT, but the differences generally narrowed over time. This can be explained the fact that the LPCa patients underwent rehabilitation treatment, such as pelvic floor muscle exercise or biofeedback therapy to achieve functional recovery.^86^, ^87^, ^88^, ^89^, ^90^, ^91^, ^92^, ^93^ One of the main mechanisms by which pelvic floor muscle training improves urinary control is the activation and coordination of the pelvic floor muscles to strengthen strength and endurance, thereby amplifying urethral pressure. RCT had shown that pelvic floor muscle exercise was effective in improving urinary control time.^94^ In addition, adaptive changes in dietary habits also can help with recovery of function.^95^, ^96^, ^97^ For example, foods rich in vitamins C and D were recommended over caffeine or alcohol.

Also worth mentioning are the unpublished but valuable results of PACE A trial (NCT01584258). PACE A was designed to determine whether HRQoL improved after stereotactic body radiotherapy compared with surgery. Consistent with the findings in this RCT, stereotactic body radiotherapy was associated with better scores on urinary incontinence and sexual bother and worse scores on the bowel subdomain than surgery after 2 years. We look forward to more RCTs comparing the effects of RT and surgery on HRQoL of LPCa in the future in order to improve the quality of meta analysis.

This meta‐analysis synthesized previous relevant clinical studies and evaluated various subgroups in the three domains of urology, bowel and sex, and refine at different time points, and found that the differences between the two groups are gradually narrowing over time. The limitations of this paper are the lack of RCTs and the inclusion of a large number of observational studies, including retrospective studies, resulting in poor matching of baseline conditions between studies. Factors such as age, tumor size and stage can affect HRQoL. For example, the function of each system itself declines with age. The larger the tumor, the greater the scope of surgery and RT, which also leads to increased damage. In addition, due to the difference of race, the scores of HRQoL is autonomous, and there are certain cognitive differences and subjectivity, leading to the difference in scores. Surgical procedures, including nerve‐sparing and non‐nerve‐sparing surgery, and RT, including internal and external irradiation, are potential factors that can affect the HRQoL.

## 5 CONCLUSION

The results of this study suggest that for LPCa, surgery generally leads to better bowel quality of life and higher urinary irritative/obstructive scores than RT. However, RT has better urinary scores, urinary function scores, urinary incontinence scores and quality of life in sexual domain. It's worth noting that the difference in HRQoL between surgery and RT diminished over time. High‐quality RCTs from multiple centers are expected to verify the findings of this study.

## AUTHOR CONTRIBUTIONS

**Chao Yu:** Investigation (equal); methodology (equal); project administration (equal); resources (equal); software (equal); writing – original draft (equal); writing – review and editing (equal). **Jie Yao:** Conceptualization (equal); data curation (equal); resources (equal); software (equal); writing – original draft (equal). **Yujing He:** Investigation (equal); resources (equal); software (equal); writing – original draft (equal); writing – review and editing (equal). **Jianing Huang:** Formal analysis (equal); software (equal); supervision (equal); writing – original draft (equal); writing – review and editing (equal). **Meiling Chen:** Conceptualization (equal); investigation (equal); methodology (equal); validation (equal); writing – original draft (equal). **Mingxia Qian:** Resources (equal); software (equal); writing – original draft (equal); writing – review and editing (equal). **Dandi Lou:** Resources (equal); software (equal); supervision (equal); writing – original draft (equal); writing – review and editing (equal). **Zhizhen Zhou:** Writing – original draft (equal); writing – review and editing (equal). **Feng Chen:** Conceptualization (equal); data curation (equal); methodology (equal); supervision (equal); writing – original draft (equal).

## FUNDING INFORMATION

The authors received no financial support in conducting this meta‐analysis.

## CONFLICT OF INTEREST STATEMENT

The authors declare no conflict of interest.

## Supporting information


Table S1.

Table S2.

Table S3.

## Data Availability

Data supporting findings reported in this study are available in the supplementary materials.
