# Supplementary material for: Effects of surgery versus radiotherapy in patients with localized prostate cancer in terms of urinary, bowel, and sexual domains
Source: Cancer Med. 2023 Jul 30;12(17):18176–88. doi: 10.1002/cam4.6395 (PMC10524086; doi:10.1002/cam4.6395)
Supplement: Supplementary file 1 — Table S1. [file CAM4-12-18176-s001.docx]

**Supplementary Table 1. Search Strategy.**

**Data searched from PubMed**

| Search Query [Title/Abstract] | Results |
| --- | --- |
| #1 “external conformal radiotherapy” OR “EBRT” OR “External beam radiation therapy” OR “brachytherapy” OR “BT” OR “Radiotherapy” OR “Radiotherapies” OR “Radiation Therapies” OR “Radiation Therapy” OR “Radiation Treatments” OR “Radiotherapies, Targeted” OR “Radiotherapy, Targeted” OR “Targeted Radiotherapies” OR “Targeted Radiotherapy” OR “Targeted Radiation Therapy” OR “Radiation Therapies, Targeted” OR “Targeted Radiation Therapies” OR “Therapies, Targeted Radiation” OR “Therapy, Targeted Radiation” OR “Radiation Therapy, Targeted” | 389,826 |
| #2 “Locally advanced prostate cancer” OR “LAPC” OR “Prostatic Neoplasms” OR “Prostate Neoplasms” OR “Neoplasms, Prostate” OR “Neoplasms, Prostatic” OR “Prostate Cancers” OR “Cancers, Prostate” OR “Cancer of the Prostate” OR “Prostatic Cancers” | 112,916 |
| #3 “Surgery” OR “Operative Procedures” OR “Surgical Procedure, Operative” OR “operations” OR “Prostatectomy” OR “Prostatectomies” OR “Prostatectomy, Suprapubic” OR “Prostatectomies, Suprapubic” OR “Suprapubic Prostatectomies” OR “Suprapubic Prostatectomy” OR “Prostatectomy, Retropubic” OR “Prostatectomies, Retropubic” OR “Retropubic Prostatectomies” OR “Retropubic Prostatectomy” | 2989,593 |
| #4 #1 AND #2 AND #3 | 3,840 |

Searched on February 1, 2023. Results: 3,840.

**Data searched from Web of Science**

| Search Query [AB, TI, KP] | Results |
| --- | --- |
| #1 “external conformal radiotherapy” OR “EBRT” OR “External beam radiation therapy” OR “brachytherapy” OR “BT” OR “Radiotherapy” OR “Radiotherapies” OR “Radiation Therapies” OR “Radiation Therapy” OR “Radiation Treatments” OR “Radiotherapies, Targeted” OR “Radiotherapy, Targeted” OR “Targeted Radiotherapies” OR “Targeted Radiotherapy” OR “Targeted Radiation Therapy” OR “Radiation Therapies, Targeted” OR “Targeted Radiation Therapies” OR “Therapies, Targeted Radiation” OR “Therapy, Targeted Radiation” OR “Radiation Therapy, Targeted” | 1274,907 |
| #2 “Locally advanced prostate cancer” OR “LAPC” OR “Prostatic Neoplasms” OR “Prostate Neoplasms” OR “Neoplasms, Prostate” OR “Neoplasms, Prostatic” OR “Prostate Cancers” OR “Cancers, Prostate” OR “Cancer of the Prostate” OR “Prostatic Cancers” | 310,428 |
| #3 “Surgery” OR “Operative Procedures” OR “Surgical Procedure, Operative” OR “operations” OR “Prostatectomy” OR “Prostatectomies” OR “Prostatectomy, Suprapubic” OR “Prostatectomies, Suprapubic” OR “Suprapubic Prostatectomies” OR “Suprapubic Prostatectomy” OR “Prostatectomy, Retropubic” OR “Prostatectomies, Retropubic” OR “Retropubic Prostatectomies” OR “Retropubic Prostatectomy” | 3721,221 |
| #4 #1 AND #2 AND #3 | 6,268 |

Searched on February 1, 2023. Results: 6,268.

**Data searched from** **Embase**

| Search Query [ab, ti] | Results |
| --- | --- |
| #1 “external conformal radiotherapy” OR “EBRT” OR “External beam radiation therapy” OR “brachytherapy” OR “BT” OR “Radiotherapy” OR “Radiotherapies” OR “Radiation Therapies” OR “Radiation Therapy” OR “Radiation Treatments” OR “Radiotherapies, Targeted” OR “Radiotherapy, Targeted” OR “Targeted Radiotherapies” OR “Targeted Radiotherapy” OR “Targeted Radiation Therapy” OR “Radiation Therapies, Targeted” OR “Targeted Radiation Therapies” OR “Therapies, Targeted Radiation” OR “Therapy, Targeted Radiation” OR “Radiation Therapy, Targeted” | 826,344 |
| #2 “Locally advanced prostate cancer” OR “LAPC” OR “Prostatic Neoplasms” OR “Prostate Neoplasms” OR “Neoplasms, Prostate” OR “Neoplasms, Prostatic” OR “Prostate Cancers” OR “Cancers, Prostate” OR “Cancer of the Prostate” OR “Prostatic Cancers” | 27,079 |
| #3 “Surgery” OR “Operative Procedures” OR “Surgical Procedure, Operative” OR “operations” OR “Prostatectomy” OR “Prostatectomies” OR “Prostatectomy, Suprapubic” OR “Prostatectomies, Suprapubic” OR “Suprapubic Prostatectomies” OR “Suprapubic Prostatectomy” OR “Prostatectomy, Retropubic” OR “Prostatectomies, Retropubic” OR “Retropubic Prostatectomies” OR “Retropubic Prostatectomy” | 6,163,053 |
| #4 #1 AND #2 AND #3 | 1,837 |

Searched on February 1, 2023. Results: 1,837.

**Data searched from Cochrane Library**

| Search Query [ti, ab, kw] | Results |
| --- | --- |
| #1 “external conformal radiotherapy” OR “EBRT” OR “External beam radiation therapy” OR “brachytherapy” OR “BT” OR “Radiotherapy” OR “Radiotherapies” OR “Radiation Therapies” OR “Radiation Therapy” OR “Radiation Treatments” OR “Radiotherapies, Targeted” OR “Radiotherapy, Targeted” OR “Targeted Radiotherapies” OR “Targeted Radiotherapy” OR “Targeted Radiation Therapy” OR “Radiation Therapies, Targeted” OR “Targeted Radiation Therapies” OR “Therapies, Targeted Radiation” OR “Therapy, Targeted Radiation” OR “Radiation Therapy, Targeted” | 43,712 |
| #2 “Locally advanced prostate cancer” OR “LAPC” OR “Prostatic Neoplasms” OR “Prostate Neoplasms” OR “Neoplasms, Prostate” OR “Neoplasms, Prostatic” OR “Prostate Cancers” OR “Cancers, Prostate” OR “Cancer of the Prostate” OR “Prostatic Cancers” | 18,440 |
| #3 “Surgery” OR “Operative Procedures” OR “Surgical Procedure, Operative” OR “operations” OR “Prostatectomy” OR “Prostatectomies” OR “Prostatectomy, Suprapubic” OR “Prostatectomies, Suprapubic” OR “Suprapubic Prostatectomies” OR “Suprapubic Prostatectomy” OR “Prostatectomy, Retropubic” OR “Prostatectomies, Retropubic” OR “Retropubic Prostatectomies” OR “Retropubic Prostatectomy” | 304,213 |
| #4 #1 AND #2 AND #3 | 870 |

Searched on February 1, 2023. Results: 870.
