# Supplementary material for: Effects of surgery versus radiotherapy in patients with localized prostate cancer in terms of urinary, bowel, and sexual domains
Source: Cancer Med. 2023 Jul 30;12(17):18176–88. doi: 10.1002/cam4.6395 (PMC10524086; doi:10.1002/cam4.6395)
Supplement: Supplementary file 2 — Table S2. [file CAM4-12-18176-s003.doc]

Supplementary Table 2. Quality assessment of studies included.

The RCTs and observational studies were assessed by the Cochrane Collaboration’s tool and Newcastle-Ottawa Quality Assessment Scale, respectively.

| Author, year,  Study (RCT) | Sequence  Generation | | Allocation  Concealment | | Blinding | Incomplete  outcome data | | Selective  outcome reporting | | Free of  other bias |  | | | | |
| --- | --- | --- | --- | --- | --- | --- | --- | --- | --- | --- | --- | --- | --- | --- | --- |
| Donovan, J L, 2016 | low risk | | low risk | | high risk | low risk | | low risk | | unclear risk |  | | | | |
|  | | | | | | | | | | |  | | | | |
| Author, year,  Study (Observational) | | **Selection (Out of 4)** | | | | | | | | | **Comparability**  **(Out of 2)** | **Outcomes (Out of 3)** | | | **Total**  **(Out of 9)** |
|  |  | Representativeness of exposed cohort | | Selection of nonexposed cohort | | | Ascertainment  of exposure | | Outcome not present at the start of the study | |  | Assessment of outcomes | Length of follow-up | Adequacy of follow-up |  |
| Downs, T M, 2003 | | 1 | | 1 | | | 1 | | 1 | | 2 | 0 | 1 | 1 | 8 |
| Namiki, S, 2004 | | 1 | | 1 | | | 1 | | 1 | | 1 | 0 | 1 | 1 | 7 |
| Namiki, S, 2006 | | 1 | | 0 | | | 1 | | 1 | | 2 | 0 | 1 | 1 | 7 |
| Namiki, S, 2010 | | 1 | | 1 | | | 0 | | 1 | | 2 | 0 | 1 | 1 | 7 |
| Miller, D C, 2005 | | 1 | | 1 | | | 1 | | 1 | | 1 | 1 | 1 | 0 | 7 |
| Symon, Z, 2006 | | 1 | | 1 | | | 1 | | 1 | | 2 | 1 | 1 | 0 | 8 |
| Korfage, I J, 2005 | | 1 | | 1 | | | 1 | | 1 | | 2 | 0 | 1 | 1 | 8 |
| Jayadevappa, R, 2006 | | 1 | | 1 | | | 1 | | 1 | | 1 | 0 | 1 | 1 | 7 |
| Hashine, K, 2008 | | 1 | | 1 | | | 1 | | 1 | | 2 | 0 | 1 | 1 | 8 |
| Hashine, K, 2009 | | 1 | | 1 | | | 1 | | 1 | | 2 | 1 | 1 | 1 | 9 |
| Ferrer, M, 2008 | | 1 | | 1 | | | 1 | | 1 | | 2 | 1 | 1 | 1 | 9 |
| Krahn, M D, 2009 | | 1 | | 1 | | | 1 | | 1 | | 2 | 0 | 1 | 1 | 8 |
| Takizawa, I, 2009 | | 1 | | 1 | | | 1 | | 1 | | 2 | 0 | 1 | 0 | 7 |
| Smith, D P, 2009 | | 1 | | 1 | | | 1 | | 1 | | 2 | 0 | 1 | 0 | 7 |
| Egger, S J, 2018 | | 1 | | 1 | | | 1 | | 1 | | 1 | 1 | 1 | 0 | 7 |
| Rice, K, 2010 | | 1 | | 1 | | | 1 | | 1 | | 2 | 1 | 1 | 1 | 9 |
| Dragićević, S, 2010 | | 1 | | 1 | | | 1 | | 1 | | 2 | 1 | 1 | 1 | 9 |
| Crook, J M, 2011 | | 1 | | 1 | | | 1 | | 1 | | 1 | 1 | 1 | 0 | 7 |
| Tol-Geerdink, J J, 2013 | | 1 | | 1 | | | 1 | | 1 | | 2 | 1 | 1 | 0 | 8 |
| Shinohara, N, 2013 | | 1 | | 1 | | | 1 | | 1 | | 2 | 0 | 1 | 1 | 8 |
| Chang, P, 2017 | | 1 | | 1 | | | 1 | | 1 | | 2 | 1 | 1 | 0 | 8 |
| Sciarra, A, 2018 | | 1 | | 1 | | | 1 | | 1 | | 1 | 0 | 1 | 1 | 7 |
| Hoffman, K E, 2020 | | 1 | | 1 | | | 1 | | 1 | | 1 | 1 | 1 | 1 | 8 |
| Ng, C F, 2020 | | 1 | | 1 | | | 1 | | 1 | | 1 | 1 | 1 | 0 | 7 |
| Wang, F, 2022 | | 1 | | 1 | | | 1 | | 1 | | 2 | 1 | 1 | 1 | 9 |
| Tiruye, T, 2022 | | 1 | | 1 | | | 0 | | 1 | | 2 | 1 | 1 | 0 | 7 |
| Taylor, K L, 2012 | | 1 | | 1 | | | 0 | | 1 | | 1 | 1 | 1 | 0 | 6 |

Risk of bias was assessed as “low risk”, “high risk” or “unclear risk”.
