# Supplementary material for: Effects of surgery versus radiotherapy in patients with localized prostate cancer in terms of urinary, bowel, and sexual domains
Source: Cancer Med. 2023 Jul 30;12(17):18176–88. doi: 10.1002/cam4.6395 (PMC10524086; doi:10.1002/cam4.6395)
Supplement: Supplementary file 3 — Table S3. [file CAM4-12-18176-s002.docx]

**Supplementary Table 3.** Characteristics of included studies in the meta-analysis of the difference of HRQoL between surgery and radiotherapy for LPCa.

| Author, year | Data source | Treatment details |
| --- | --- | --- |
| Downs, T M, 2003 | CaPSURE (Cancer of the Prostate Strategic Urological Research Endeavor) data base | BT: interstitial seed implantation (103palladium, 125iodine, 192Iridium) |
| Namiki, S, 2004 | Tohoku University hospital, Miyagi Cancer Center, Sendai Shakaihoken Hospital, Kurashiki Central Hospital, Furukawa City Hospital | EBRT |
| Namiki, S, 2006 | Tohoku University Hospital and Kitasato University Hospital | BT: I-125 |
| Namiki, S, 2010 | Tohoku University Hospital, Miyagi Cancer Center | EBRT: 3D-CRT (70 Gy) or IMRT (78 Gy) |
| Miller, D C, 2005 | Michigan Urology Center and Department of Radiation Oncology, University of Michigan Medical Center, Beth Israel-Deaconess Medical Center, Harvard Medical School | EBRT: 3D-CRT  BT |
| Symon, Z, 2006 | multidisciplinary urologic oncology clinic | EBRT |
| Korfage, I J, 2005 | University Medical Center Rotterdam; St. Franciscus Gasthuis; Medical Center Rotterdam Zuid, locations ‘‘Clara’’ and ‘‘Zuider’’ | EBRT: an average of 33 radiation sessions over 7 weeks |
| Jayadevappa, R, 2006 | an urban academic and a Veterans’ Administration (VA) hospital | EBRT |
| Hashine, K, 2008 | Urology and Radiation Oncology, National Hospital Organization Shikoku Cancer Center, Matsuyama, Japan | BT: 145 Gy to the prostate with an I-125 |
| Hashine, K, 2009 | Urology and Radiation Oncology, National Hospital Organization Shikoku Cancer Center Matsuyama, Japan | BT: 145 Gy to the prostate with an I-125 |
| Ferrer, M, 2008 | 10 Spanish hospitals (two urology and eight radiation Oncology departments) | EBRT: 1.8 to 2.0-Gy daily fractions, 5 days per week, to a mean (SD) dose of 74.03 (4.3) Gy  BT: 144 Gy to the prostate with an I-125 |
| Krahn, M D, 2009 | clinics of urologists and radiation oncologists at the Princess Margaret Hospital | RT |
| Takizawa, I, 2009 | Urology and Radiology, Niigata University Hospital (Niigata, Japan) | EBRT: 3D-CRT (70.0-71.0 Gy) |
| Smith, D P, 2009 | New South Wales central cancer registry | EBRT, BT |
| Egger, S J, 2018 | New South Wales central cancer registry | BT: low dose-rate brachytherapy |
| Rice, K, 2010 | Urology Service, Department of Surgery, Walter Reed Army Medical Center; Center for Prostate Disease Research | EBRT: 3D-CRT with 2.0-Gy daily fractions administered 5 days per week until a total dose of 76 Gy  BT: 160 Gy to the prostate with an I-125 |
| Dragićević, S, 2010 | Institute of Epidemiology, School of Medicine, University of Belgrade, Belgrade, Serbia | BT: 145 Gy to the prostate with an I-125 |
| Crook, J M, 2011 | The Surgical Prostatectomy Versus Interstitial Radiation Intervention Trial (SPIRIT) | BT |
| van Tol-Geerdink, J J, 2013 | Radboud University Nijmegen Medical Centre, the Canisius Wilhelmina Hospital in Nijmegen, and the Rijnstate Hospital in Arnhem. | EBRT: IMRT (78 Gy)  BT: low-dose seed implants |
| Shinohara, N, 2013 | Renal and Genitourinary Surgery, Hokkaido University Graduate School of Medicine | EBRT: IMRT (75 Gy) |
| Donovan, J L, 2016 | ProtecT trial (NCT02044172) | EBRT: 3D-CRT (74 Gy) |
| Chang, P, 2017 | The Prostate Cancer Outcomes and Satisfaction with Treatment Assessment (PROST-QA) | EBRT: IMRT or 3D-CRT  BT: low-dose rate I-125 |
| Sciarra, A, 2018 | Urological Sciences, University Sapienza, Rome, Italy | EBRT: IMRT (75 Gy ) or 3D-CRT |
| Hoffman, K E, 2020 | 5 population-based Surveillance, Epidemiology and End Results Program registries and the observational Cancer of the Prostate Strategic Urologic Research Endeavor prostate cancer registry | EBRT  BT: low-dose-rate |
| Ng, C F, 2020 | Urology Centre, Department of Surgery, The Chinese University of Hong Kong | EBRT |
| Wang, F, 2022 | Northern Jiangsu People’s Hospital | BT: 145 Gy to the prostate with an I-125 |
| Tiruye, T, 2022 | South Australian Prostate Cancer Clinical Outcomes Collaborative (SA-PCCOC) database | EBRT, BT |
| Taylor, K L, 2012 | US-based Prostate, Lung, Colorectal, and Ovarian Cancer Screening Trial (PLCO) | RT |

LPCa, localized prostate cancer; EBRT, external beam radiotherapy; BT, brachytherapy; 3D-CRT, 3 dimensional conformal radiation therapy; IMRT, intensity modulated radiation therapy; RT, radiotherapy; SD, standard deviation; HRQoL, health-related quality of life.
